# Development of an application for providing corneal topography reports based on artificial intelligence

**DOI:** 10.5935/0004-2749.20220051

**Published:** 2025-08-21

**Authors:** Abrahão Rocha Lucena, Mariana Oliveira de Araújo, Rômulo Férrer Lima Carneiro, Tarique da Silveira Cavalcante, Alyson Bezerra Nogueira Ribeiro, Francisco José Marques Anselmo

**Affiliations:** 1 Escola Cearense de Oftalmologia, Fortaleza, CE, Brazil; 2 Instituto Federal de Educação, Ciência e Tecnologia do Ceará, Fortaleza, CE, Brazil; 3 Universidade Federal do Ceará, Fortaleza, CE, Brazil

**Keywords:** Mobile, Artificial intelligence, Corneal topography, Astigmatism, Dispositivos móveis, Inteligência artificial, Topografia corneana, Astigmatismo

## Abstract

**Purpose:**

To develop an application (*TopEye*) in the iOS platform for mobile devices to allow the capture and interpretation of color maps generated by corneal topographers using artificial intelligence.

**Methods:**

In the execution, follow-up, and assessment of the project, we used the Scrum methodology and interactive and incremental development process for the project management and agile software development. The ge nerated diagnostic pattern bank consists of 1,172 examples of corneal topography, divided into 275 spherical, 302 symmetrical, 295 asymmetrical, and 300 irregular patterns (keratoconus). For the development of the artificial intelligence of the application, network training was established with 240 images of each pattern type, with a total of 960 patterns (81.91%). The remaining 212 images (18.09%) were used to test the application and will be used for the results. The process is semi-automatic, so the topographic image is captured with a smartphone, the examiner performs the contour of the corneal relief manually, and then the neural network performs the diagnosis.

**Results:**

The application diagnosed 201 cases (94.81%) correctly. In 212 images, the algorithm missed the classification of 11 cases (5.19%). The major error that occurred was in distinguishing between symmetrical and asymmetrical classes. In keratoconus screening, the application reached 95.00% sensitivity and 98.68% specificity.

**Conclusion:**

The work resulted in obtaining an efficient application to capture topographic images using a smartphone camera and their interpretations through applied artificial intelligence.

## Abstract (pt)

**Objetivo:**

Desenvolver um aplicativo (*TopEye*) na plataforma iOS para dispositivos móveis que possibilite a captação e interpretação do mapa de cores gerados por qualquer topógrafo corneano através da inteligência artificial (IA).

**Métodos:**

A execução, acompanhamento e avaliação do projeto foi utilizada a metodologia Scrum, processo de desenvolvimento interativo e incremental para gerenciamento de projetos e desenvolvimento ágil de software. O banco de padrões de diagnóstico gerado consiste em 1172 exemplos, divididos em: 275 padrões esféricos, 302 regulares simétricos, 295 regulares assimétricos e 300 irregulares (ceratocone). Para o desenvolvimento da inteligência artificial do aplicativo, foi estabelecido o treinamento da rede com 240 imagens de cada tipo de padrão, totalizando 960 (81,91%) padrões. O restante das imagens, 212 (18,09%), foram utilizadas para testar o aplicativo e usadas para gerar os resultados. O processo é semiautomático, assim a captação da imagem topográfica é realizada com smartphone, o examinador realiza o contorno do relevo corneano manualmente para em seguida a rede neural realizar o diagnóstico.

**Resultados:**

O aplicativo diagnosticou 201 (94,81%) imagens corretamente. De um total de 212 imagens, o algoritmo errou a classificação de apenas 11 (5,19%). A principal ocorrência de erro foi na distinção das classes simétrica e assimétrica. No rastreio do ceratocone o aplicativo alcançou 95,00% de sensibilidade e 98,68% especificidade.

**Conclusão:**

O trabalho resultou na obtenção de um aplicativo eficiente na captura da imagem topográfica pela câmera do smartphone e na interpretação da mesma através da inteligência artificial aplicada.

## INTRODUCTION

Physicians in the field of ophthalmology have identified the need to create an application that can provide reports of an examination widely used in the corneal topography area. Videokeratography or corneal topography is an excellent tool for screening keratoconus, even when the disease signs are not yet evident in the slit lamp^(1-4)^. Reports can be issued through the subjective interpretation of topographic images acquired from the corneal surface^(5,6)^. Hence, the process is vulnerable to doubts and disagreements among several professionals. In addition, the learning curve is steep until the physician feels able to interpret the different existing patterns.

Several numerical indexes have already been developed^(6-8)^ that automatically classify the corneal surface according to the presence and type of astigmatism, besides suggesting the presence of keratoconus, but they still lack sensitivity and specificity^(6,9)^, thus making the diagnosis of subtle changes in the corneal surface unsafe. Another disadvantage is the investment required to purchase the specific software fosar the topography equipment^(10)^.

By combining such challenges with the market trends focused on health interactivity with technology, represented by smartphones in this proposal, we felt motivated to develop an application capable of identifying the presence of astigmatism on the corneal surface through a neural network powered by an experienced physician, who transferred his personal experience to the subjective recognition of the topographic patterns to the application.

The objective of the present study was to develop an application for smartphones in the iOS platform (*TopEye*) by using artificial intelligence (AI) for capturing and diagnosing the topographic images generated by topographers through the smartphone screen outline, thereby facilitating the elaboration of a final report and enabling the dissemination of medical knowledge concerning the examination.

## METHODS

The methodology applied for the execution, follow-up, and assessment of the project was Scrum, which is an interactive and incremental development process for the project management and agile software development.

**Figure 1:**
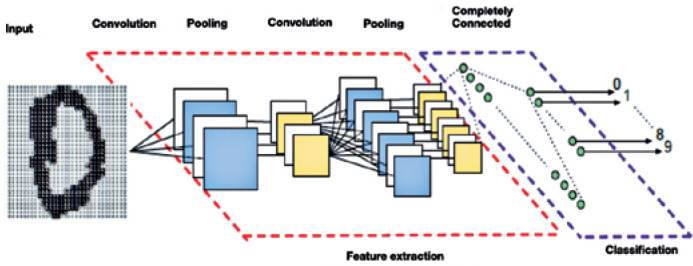
Convolutional neural network architecture.

The adopted strategy was the use of the convolutional neural network (CNN), an AI algorithm used to classify the presence of pathologies in the eye. This type of network uses a hierarchical system that tries to represent the structure in relation to the recognition of an image, where pixels form edges, edges form patterns, patterns form objects, which in turn describe the scenes. The CNN consists of one or more pairs of convolution and max-pooling layers (Figure 1)^(11)^.

Convolutional layers apply filters that process small parts of an image and are replicated throughout the image. The max-pooling layers generate a lower-resolution version of the convolution layers by applying the maximum activation of the filter in several positions within a window. Thus, more tolerance is added for specific regions of a given object in the image. The higher layers use filters that work from low-resolution inputs to process the most complex parts of the image. Each layer has a set of filters, also known as the kernel, that is responsible for extracting local features from an input. Thereby, several convolution and pooling maps can be created, containing several specific characteristics such as borders, color intensity, contours, and shapes. Each map of characteristics has a set of shared weights, which reduces the computational complexity of the network^(11)^.

The specific objective of this work was to assist the diagnosis of four existing clinical patterns in corneal topography images as follows: spherical cornea, cornea with regular symmetrical astigmatism, cornea with regular asymmetrical astigmatism, and cornea with irregular astigmatism (keratoconus).

**Figure 2:**
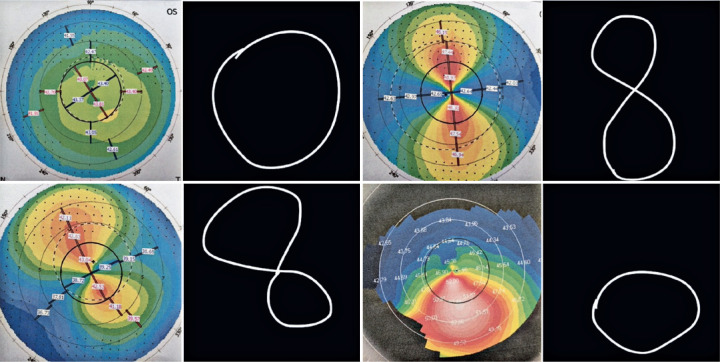
Upper panels: a spherical cornea (left) and cornea with regular symmetrical astigmatism (right). Lower panels: a cornea with regular asymmetrical astigmatism (left) and cornea with an irregular astigmatism or keratoconus (right).

The response of the algorithm depends on the manual intervention of the operator, so the application works semi-automatically. Thus, a specific contour must be created manually according to the application tutorial. The neural network will then classify the contour patterns. Figure 2 shows some manually created contours that represent the topographic patterns defined by the specialist physician.

**Figure 3:**
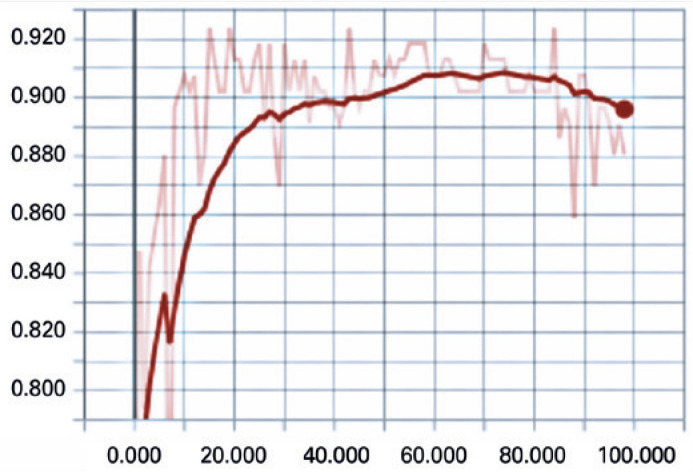
Network success rate in 100 training seasons for images with regular and symmetrical astigmatism.

The diagnostic pattern bank generated by the specialist physician consists of 1,172 examples, divided into 275 spherical patterns, 302 regular symmetrical astigmatism patterns, 295 regular asymmetrical astigmatism patterns, and 300 irregular astigmatism patterns (keratoconus). For the development of the AI of the application, network training was established with 240 images of each diagnostic pattern type, with a total of 960 training patterns (81.91%). The remaining 212 images (18.09%) were used to test the application and used for the results.Figure 3 displays the behavior of the network showing an average of 90% hit with the test set.

**Figure 4:**
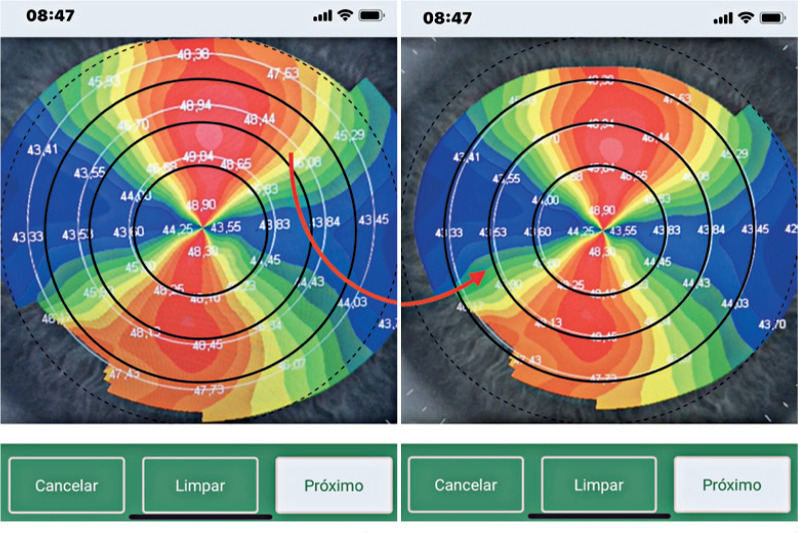
The 3-, 5-, and 7-mm zones of the application must coincide with the same zones of the corneal topography.

After opening the application, the examiner clicks on the Capture Image button; centers the printed topographic image (thermogram) on the smartphone screen, coinciding the 3-, 5-, and 7-mm zones of the topographic image with the 3-, 5-, and 7-mm zones of the application; and finally, clicks on the smartphone central screen (Figure 4).

The images acquired in RGB (red, green, and blue) are transformed into HSV (hue, saturation, and value) for better image quality. Starting from the definition of the four points that represent the vertex of the input image with possible distortion, a two-dimensional convolution is performed to define the homographic transform. To obtain the diagnosis, the examiner uses the index finger to outline the corneal area that characterizes the typical patterns of the corneal surfaces, as shown below^(12)^.

**Figure 5:**
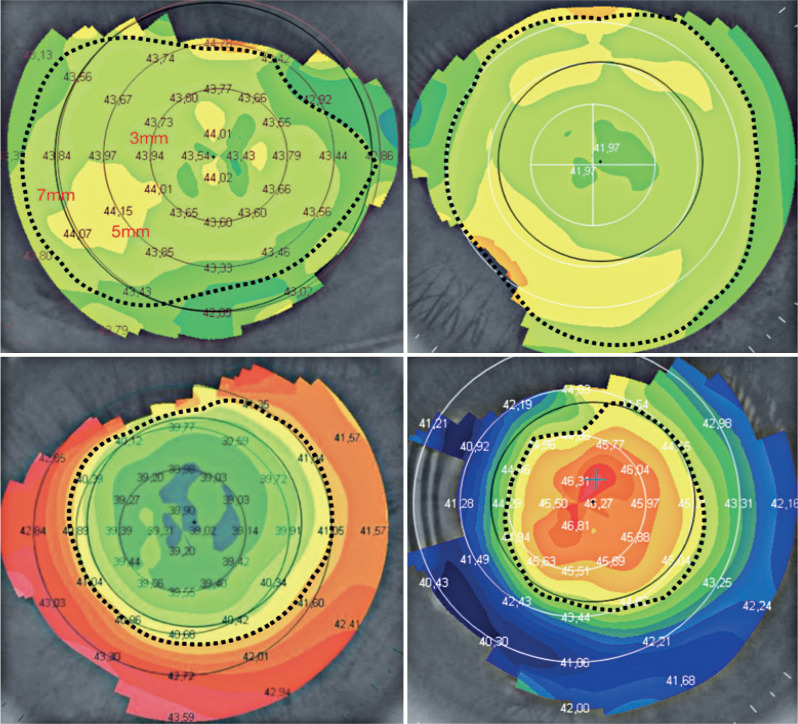
Upper panels: contours of a spherical surface. Lower panels: spherical surface contours in myopia treated with laser refractive surgery (left) and spherical surface contours in hyperopia treated with laser refractive surgery (right).

The spherical corneas have similar curvature radii. The central topographic areas of 3-, 5-, and 7-mm present similar color patterns in the green variant, without a defined tie pattern. The examiner draws a peripheral outline, as shown in the black dotted line (Figure 5, upper right and left). A differential diagnosis is performed with the surfaces submitted to laser refractive surgery for myopia (Figure 4, bottom left) or hyperopia (Figure 4, bottom right), which may also have their central areas with a spherical design. In those cases, the surface submitted to surgery for myopia shows a central area with colder colors (green or blue variants) and a central area with warmer colors (yellow or red) for hyperopia.

**Figure 6:**
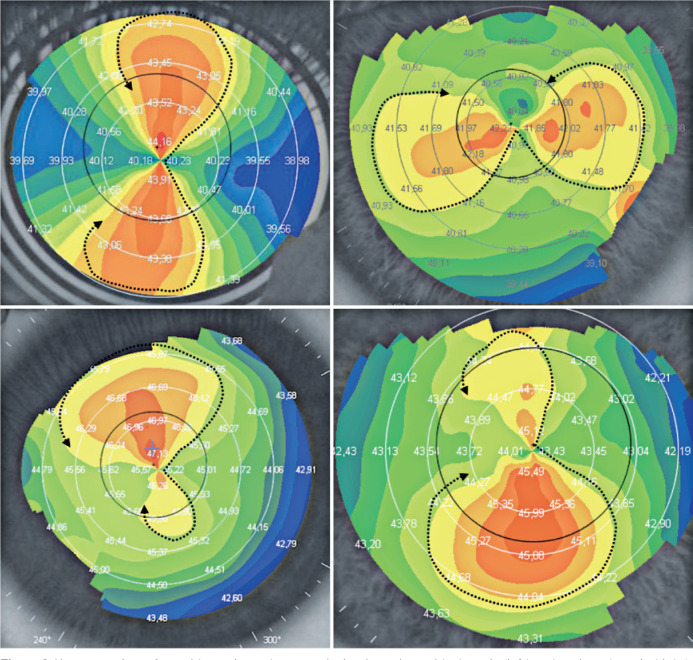
Upper panels: surface with regular and symmetrical astigmatism, with-the-rule (left) and against-the-rule (right). Lower panels: surfaces with regular astigmatism with superior asymmetry (left) and inferior asymmetry (right).

**Figure 7:**
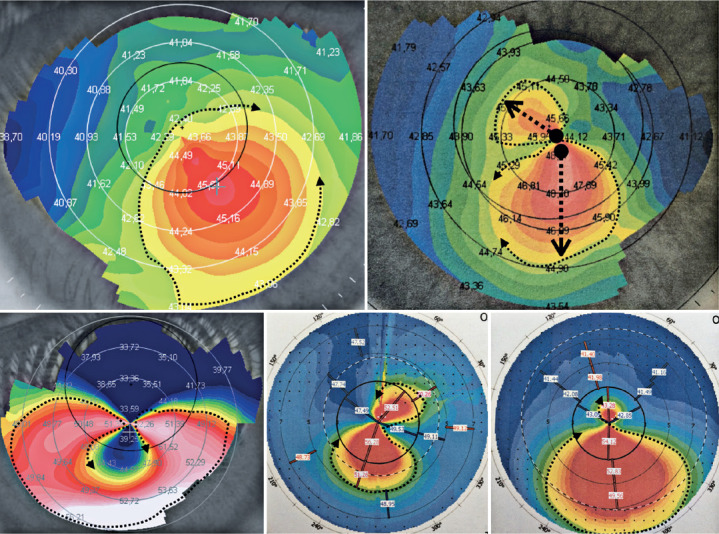
Irregular astigmatisms (keratoconus): increased classic inferior curvature (upper left), loss of perpendicularity (upper right), “pellucid marginal degeneration” (lower left), incomplete bow tie (lower central), and increased inferior curvature with a superior rudimentary tie (lower right).

In the case of corneal astigmatism, the surface was divided into two types as follows:

- Regular astigmatism: The corneal surface was divided into two meridians, the flattest and the most curved, forming an approximate angle of 90°. Regular astigmatism was further subdivided into symmetrical and asymmetrical. Regular astigmatism shows a “bow tie” formation in the more curved meridian. The tie can be made up of two halves (semimeridians) of similar sizes (Figure 6, upper panel) or different sizes, where one of the two halves presents a different length and/or width (Figure 6, bottom). The ties are usually made up of more than one color, having several leaflets, and the examiner must outline the tie following the same color shade in the two semimeridians^(13)^.
- Irregular astigmatism or keratoconus: The corneal surface does not present well-defined meridians, but presents a surface with an increase in curvature in a specific area (usually inferior) with a flat adjacent region (Figure 7, upper left). Other classic forms of irregular astigmatism have also been cataloged, such as in the cases of perpendicularity loss between the most curved and flattest meridians (Figure 7, upper right) and pellucid marginal degeneration (Figure 7, lower left). Special cases were also recorded, including the presence of symmetry between the semimeridians with a small tie, which reaches up to the 5-mm zone (Figure 6, lower central) and the bow tie formations with a rudimentary half^(11)^ (Figure 6, lower right).

## RESULTS

Of the 212 images captured and used for the test, 201 images (94.81%) were correctly classified and 11 images (5.19%) were incorrectly classified by the algorithm (Table 1).

**Table 1:** The mistakes and successes of the contours of the corneal surface according to their reliefs

Appearance of the corneal surface	TopEye success	TopEye mistake	Total
Spherical	34 (97.15%)	01 (2.85%)	35 (100%)
Regular and symmetrical astigmatism	58 (93.55%)	04 (6.45%)	62 (100%)
Regular and asymmetrical astigmatism	52 (94.55%)	03 (5.45%)	55 (100%)
Irregular astigmatism (keratoconus)	57 (95.00%)	03 (5.00%)	60 (100%)
Total	201 (94.81%)	11 (5.19%)	212 (100%)

In the set of 35 spherical pattern images, only one mistake (2.85%) was made, which the network classified as an irregular pattern. In the set of 62 regular and symmetrical pattern images, 4 mistakes (6.45%) were made. The network mistakenly classified one of the images as an irregular pattern and the remaining 3 as asymmetrical.

In the set of 55 images with an asymmetrical pattern, 3 mistakes (5.45%) were made. The network classified it as a symmetrical pattern in all the images. In the set of 60 images with irregular patterns, 3 mistakes (5.00%) were made. The network classified it as a symmetrical pattern in 1 case and as an asymmetrical pattern in the remaining two cases.

Considering keratoconus screening, the application correctly interpreted 57 of 60 images with irregular astigmatism (sensitivity of 95.00% and 5.00% false negativity rate). Of the 152 images that were not keratoconus (spherical cornea, regular and symmetrical astigmatism, and regular and asymmetrical astigmatism), the application confirmed 150 cases (98.68% specificity and 1.32% false positives).

## DISCUSSION

The major misinterpretation of the *TopEye* application was in distinguishing the classes of regular and symmetrical astigmatisms (4 mistakes in 62 images), as shown in Table 1. The gold standard pattern created by the ophthalmologist has some examples in which the difference between the symmetrical and asymmetrical patterns was not enough for the algorithm to differentiate. Usually, the images of symmetrical and asymmetrical patterns differ exactly by the symmetry between the curves. However, the gold standard pattern includes unusual patterns, thus confusing the training of the neural network. The mistake is justified by the non-coincidence during the capture of the images from the 3, 5, and 7-mm zones of the topographic map with the equivalent application zones. Another fact is that in the suggested symmetry, the two halves of the tie reach similar areas. Significant differences in the reach of the two halves of the tie or even in the width of the base may also configure asymmetry, thus making differentiation difficult.

With the *TopEye* application, the subtle irregularity changes (keratoconus) such as the increase in the curvature in the inferior region, without the formation of the other half of the tie in the superior region, were markedly valued when the database was “fed”. Visually, the superior half of the tie appears “amputated”. The examiner may already be facing a subclinical form of irregular astigmatism or keratoconus, and the correct interpretation of that finding may protect the patient from a mistaken indication for a laser refractive surgery. With the application, differentiating the irregular pattern was difficult when the superior half of the tie was rudimentary. Some of the cases were mistakenly classified as regular and asymmetrical astigmatism, strengthening the idea of the correct alignments of the 3-, 5-, and 7-mm zones during the capture of topographic maps to minimize the mistake. The relatively small database used for testing can also be considered as justification for this error.

Regular astigmatism with inferior asymmetry by itself already conveys the idea of a cornea with low resistance. When the asymmetry presents with a great disproportion in the length of the two halves of the tie or in the diameters of the base (width), the examiner may be facing an irregular astigmatism (keratoconus). This finding already changes the physician’s conduct in relation to patient follow-up by imposing conducts aimed at delaying the evolution of the irregularity, contraindicating therapies such as the laser refractive surgery.

The evident forms of regular and symmetrical astigmatisms, classic irregular astigmatism (keratoconus), or spherical corneal pattern do not require great clinical reasoning for diagnosis by the experienced physician. Nevertheless, automated numerical indexes have provided assistance to professionals for many years in the diagnosis of changes in the corneal surface^(2,6-8)^. To access those indexes, the physician must invest a lot to purchase corneal topography equipment with those indexes, besides undergoing training aimed at interpreting those indexes, deviating from the interpretative study of the topographic image.

The *TopEye* application will be available in the iOS platform with a projected price of US$3.99. It is based exclusively on the colored reliefs generated by the corneal curvature, encouraging the examiner to be trained in the clinical interpretation of the corneal surface. Each report generated is accompanied by the “know+” tool, with texts directed to the examiner to deepen one’s theoretical knowledge about each diagnosis.

By knowing the challenges and complexities involved in the subjective interpretation of topographic images and, considering that the personal experience of a skilled professional was transferred to the neural network of the application, *TopEye* has high sensitivity and specificity, which are comparable with the numerical indexes of the corneal topography equipment available in the market^(14,15)^. If the application’s tutorial is followed, the diagnosis of topographic images no longer has a subjective characteristic, where examiners have their own interpretation, to follow a unique classification pattern based on the experience of only one examiner. The interpretations of the corneal numerical indexes remain a great challenge to examiners^(16)^, so the *TopEye* application appears as a tool not only for diagnosis but also for learning, as the reports are followed by concepts about the suggested hypothesis.

The transference of human skills to artificial neural networks in the form of software or applications is already a present reality and projects great perspectives for the near future^(17,18)^. In phase 2 of this research, we project the elimination of the need for manual contouring of topographic images on the smartphone screen and making a diagnosis based only on the differentiation between the surface colors of the topographic maps.

The *TopEye* AI application showed high reliability with an easy-to-use and low-cost platform (US$3.99). It captured cornea topographic images and, through the contour of the images visualized through the smartphone screen, diagnosed the four basic patterns, namely spherical cornea, cornea with regular and symmetrical astigmatism, cornea with regular and asymmetric astigmatism, and cornea with irregular astigmatism or keratoconus (Table 1). Thus, it facilitates the preparation of topographic reports besides disseminating knowledge on the subject through the “know+” tool or by reading the theory of contours available in its tutorial.
